# Editorial: Disease and pest resistance in rice

**DOI:** 10.3389/fpls.2023.1333904

**Published:** 2023-11-22

**Authors:** Zuhua He, Zhengguang Zhang, Giampiero Valè, Blanca San Segundo, Xuewei Chen, Janila Pasupuleti

**Affiliations:** ^1^ Center for Excellence in Molecular Plant Sciences, Chinese Academy of Sciences (CAS), Shanghai, China; ^2^ Nanjing Agricultural University, Nanjing, China; ^3^ Dipartimento per lo Sviluppo Sostenibile e la Transizione Ecologica, Università del Piemonte Orientale, Vercelli, Italy; ^4^ Department of Plant Responses to Stress, Centre for Research in Agricultural Genomics (CRAG) and Spanish National Research Council, Barcelona, Spain; ^5^ State Key Laboratory of Crop Gene Exploration and Utilization in Southwest China, Sichuan Agricultural University, Chengdu, China; ^6^ Department of Entomology, International Crops Research Institute for the Semi-Arid Tropics (ICRISAT), Hyderabad, India

**Keywords:** rice, pathogens, insects, resistance, interaction, breeding

Rice (*Oryza sativa* L.), as one of the most important crops worldwide, has been widely adopted as a model for studying cereal crops and monocot plants. Rice production is severely threatened by pathogens and pests during the entire growth season, causing an estimated 10%–30% yield loss annually (Douglas, 2018; Savary et al., 2019). Therefore, disease and pest resistance has been one of the major goals in rice breeding. However, rice improvement has encountered a bottleneck largely due to the lack of useful disease resistance (*R*) genes and molecular approaches in breeding programs. Most *R* genes have limited application because of race-specificity. Therefore, the identification and application of nove*l R* genes with broad-spectrum resistance have been a big challenge.

Among many rice diseases, those causing the most relevant yield losses include the rice fungal blast caused by *Magnaporthe oryzae* (*M. oryzae*), bacterial blight caused by *Xanthomonas oryzae* pv. *oryzae* (*Xoo*), sheath blight caused by the necrotrophic fungus *Rhizoctonia solani* (*R. solani*), false smut caused by the obligate biotrophic fungus *Ustilaginoidea virens* (Cke.) Tak (*U. virens*), and viral diseases including rice tungro virus and rice stripe virus. In addition, insect pests and plant parasitic nematodes have also caused huge yield loss of rice. Over the past 30 years, impressive advancements have been achieved in *R* gene discovery and molecular mechanisms of disease resistance and growth-defense trade-offs (Deng et al., 2020; Li et al., 2020). These achievements have greatly facilitated the breeding of new disease and pest resistance cultivars.

Within this specific topic on ‘Disease and Pest Resistance in Rice’, we aim to provide new knowledge on *R* gene discovery, mechanisms of rice immunity and resistance against pathogens and insects, and technology for rice improvement. Launched in Oct, 2021, this topic received 16 manuscripts in total and ultimately published 5 papers after peer-view, including four research articles and one review. We also regret that other submissions were not accepted for publication after reviewing.

Rice blast has been the most devastating crop fungal disease worldwide (Dean et al., 2012). During the past decade, tremendous progress has been made on molecular mechanisms of rice defense responses against *M. oryzae*. Luo et al. reported that the rice FERONIA-like receptor (FLR) is involved in resistance against *M. oryzae*. They observed enhanced susceptibility to *M. oryzae* in the *flr1* mutant, associated with the suppression of defense-related gene expression. Thus, FLR1 positively regulates blast resistance. A low concentration of Ca^2+^ induced *FLR1* expression, and the Ca^2+^ content in the *flr1*mutant was significantly lower than that in wild-type plants. Some of the differentially expressed genes (DEGs) in the *flr1* mutant revealed by RNA sequencing were found to play a role in cellular metal ion homeostasis and transition metal ion homeostasis, suggesting that FLR1 regulates Ca^2+^ homeostasis. Therefore, FLR1 mediates resistance to *M. oryzae* through modulating Ca^2+^ homeostasis. This study links FLR-mediated immune response with Ca^2+^ signaling, advancing our understanding of rice-*M. oryzae* interaction.

Most blast resistance (*Pi*) genes encode nucleotide-binding and leucine-rich repeat (NLR) receptors that trigger effector-triggered immunity (ETI) and have been widely deployed in rice blast resistance breeding (Deng et al., 2020; Li et al., 2020). *Pigm* confers broad-spectrum and durable blast resistance without affecting grain yield by balancing the effects of a pair of antagonistic genes, *PigmR* and *PigmS* (Deng et al., 2017). The Shimin Zuo laboratory at Yangzhou University developed new elite rice varieties using the *Pigm* gene (Feng et al.). Using breeding strategies such as backcross and marker-assisted selection (MAS), they introduced *Pigm* into two good-quality *japonica* cultivars, Huageng 8/HG8 and Wuyungeng 32/WYG32, to obtain advanced backcross lines (ABLs) with *Pigm*, along with ABLs containing other 13 known *Pi* gene loci. All these ABLs displayed stronger resistance both in seedling-inoculation assay using 184 isolates collected from the lower region of Yangtze River, and in panicle- inoculation assay using mixed isolates, than the corresponding recurrent parent, which was further confirmed in natural nursery trails. Of note, no change was observed in agronomically important morphological traits. One *Pigm* line Yangnonggeng 3091, with excellent performance on blast resistance and grain yield and quality, was authorized as a new commercial variety in Jiangsu province in 2021. Taken together, this study demonstrated the advantage of *Pigm* in developing new disease resistance rice cultivars without growth penalty (Feng et al.).

*Xoo*, the causal agent of bacterial leaf blight (BLB) specifically infects the vascular system through wounding and hydathodes, and colonizes in xylem vessels, resulting in systemic spread and infection of *Xoo*. Over the last three decades, more than 40 BLB resistance genes/loci (*Xa*) have been identified. In the review of Yang et al., the progress on cloning and functional study of 44 *Xa* genes is summarized. Among them, 28 were dominant and 16 were recessive. 15 genes, including *Xa1*, *Xa2*/*Xa31*, *Xa3*/*Xa26*, *Xa4*, *xa5*, *Xa7*, *Xa10*, *xa13*, *Xa14*, *Xa21*, *Xa23*, *xa25*, *Xa27*, *xa41*, and *Xa45*, have been cloned and characterized. In particular, the first cloned *Xa* gene, *Xa21*, encodes a receptor-like kinase (RLK) that triggers pattern-triggered immunity (PTI) via perceiving RaxX and activating downstream XB signaling components.

Among the *Xa* genes, only the *Xa1*/*Xa2/Xa14/Xa31/Xa45* alleles and *Xa47* encode typical NLRs (Ji et al., 2020; Lu et al., 2022; Yang et al.; Yoshimura et al., 1998). Plants have evolved the vascular-specific immunity to combat *Xoo* (Lin et al., 2022). It has been suggested that the long coevolution has shaped the race-cultivar specificity of rice-*Xoo* interactions and their reciprocal adaptation, characterized by differentiation among *Xoo* races corresponding to *indica* and *japonica* rice accessions (Zhang et al., 2021). Intriguingly, most functional *Xa* genes/loci were isolated from wild relatives or recessive *xa* mutations of susceptibility genes. However, whether these genetic phenomena reflect negative selection on the *Xa* loci keeps unknown.

The recent outbreak of other diseases threatens rice production in some regions. For instance, brown spot of rice (BSR), mainly caused by the fungal pathogens *Bipolaris oryzae* and *Exserohilum rostratum*, is threating rice production in sub-Saharan Africa. Additionally, 80% rice field is infected by BSR in western Burkina Faso. A genomic study of *Bipolaris oryzae* and *Exserohilum rostratum* that cause brown spot disease of rice in Burkina Faso revealed critical information on the biology and population structures of the two major fungi, providing a genetic basis for studying disease resistance against the pathogens (Kaboré et al.).

Rice insect pests are generally classified as chewing and piercing-sucking insects based on their feeding modes. Chewing insects such as leaf folders and stem borers directly damage plant by cutting and chewing important plant organs including leaves and stems. Piercing-sucking insects, for instance, brown planthopper (*Nilaparvata lugens*, BPH) and small brown planthopper (*Laodelphax striatellus*, SBPH), feed on vascular tissues and secrete salivary effectors into the host during feeding, causing wilting and death of rice plants (Cheng et al., 2013). Fu et al. reported that H_2_O_2_ burst and callose deposition form a common defensive mechanism against piercing-sucking insects. Calmodulin (CaM), secreted by BPH salivary gland, suppresses the accumulation of H_2_O_2_ and callose. CaM-silenced BPH and SBPH failed to penetrate the phloem. *In planta* expression of CaM suppressed while CaM-silenced BPH and SBPH nymphs promoted defense response such as H_2_O_2_ accumulation and callose deposition, adding new information to our understanding of molecular plant-insect interactions.

Our understanding of the mechanisms and signaling pathways of rice immunity is still limited, in comparison with extensive studies in the dicot model plant Arabidopsis. Exploiting rice immune signaling is critical for breeding disease resistance rice varieties to combat pathogens and insect pests. This topic on Disease and Pest Resistance in Rice certainly provides new knowledge and technology for developing more resilient rice cultivars to diseases and pests.

## Author contributions

ZH: Writing – original draft, Writing – review & editing. ZZ: Writing – original draft. GV: Writing – original draft. BS: Writing – original draft. XC: Writing – review & editing. JP: Writing – review & editing

## References

[B1] Cheng X. Zhu L. He G. (2013). Towards understanding of molecular interactions between rice and the brown planthopper. Mol. Plant 6, 621–634. doi: 10.1093/mp/sst030 23396040

[B2] Dean R. Van Kan J. A. L. Pretorius Z. A. Hammond-Kosack K. E. Di Pietro A. Spanu P. D.. (2012). The top 10 fungal pathogens in molecular plant pathology. Mol. Plant Pathol. 13, 414–430. doi: 10.1111/j.1364-3703.2011.00783.x 22471698 PMC6638784

[B3] Deng Y. Ning Y. Yang D. L. Zhai K. Wang G. L. He Z. (2020). Molecular basis of disease resistance and perspectives on breeding strategies for resistance improvement in crops. Mol. Plant 13, 1402–1419. doi: 10.1016/j.molp.2020.09.018 32979566

[B4] Deng Y. Zhai K. Xie Z. Yang D. Zhu X. Liu J.. (2017). Epigenetic regulation of antagonistic receptors confers rice blast resistance with yield balance. Science 355, 962–965. doi: 10.1126/science.aai8898 28154240

[B5] Douglas A. E. (2018). Strategies for enhanced crop resistance to insect pests. Annu. Rev. Plant Biol. 69, 637–660. doi: 10.1146/annurev-arplant-042817-040248 29144774

[B6] Ji C. Ji Z. Liu B. Cheng H. Liu H. Liu S.. (2020). Xa1 allelic R genes activate rice blight resistance suppressed by interfering TAL effectors. Plant Commun. 1 (4), 100087. doi: 10.1016/j.xplc.2020.100087 33367250 PMC7748017

[B7] Li W. Deng Y. Ning Y. He Z. Wang G. L. (2020). Exploiting broad-spectrum disease resistance in crops: from molecular dissection to breeding. Annu. Rev. Plant Biol. 71, 575–603. doi: 10.1146/annurev-arplant-010720-022215 32197052

[B8] Lin H. Wang M. Y. Chen Y. Nomura K. Hui S. G. Gui J. S.. (2022). An MKP-MAPK protein phosphorylation cascade controls vascular immunity in plants. Sci. Adv. 8 (10), eabg8723. doi: 10.1126/sciadv.abg8723 35263144 PMC8906744

[B9] Lu Y. Zhong Q. Xiao S. Wang B. Ke X. Zhang Y.. (2022). A new NLR disease resistance gene *Xa47* confers durable and broad-spectrum resistance to bacterial blight in rice. Front. Plant Sci. 13, 1037901. doi: 10.3389/fpls.2022.1037901 36507384 PMC9730417

[B10] Savary S. Willocquet L. Pethybridge S. J. Esker P. McRoberts N. Nelson A. (2019). The global burden of pathogens and pests on major food crops. Nat. Ecol. Evol. 3, 430–439. doi: 10.1038/s41559-018-0793-y 30718852

[B11] Yoshimura S. Yamanouchi U. Katayose Y. Toki S. Wang Z. X. Kono I.. (1998). Expression of *Xa1*, a bacterial blight-resistance gene in rice, is induced by bacterial inoculation. Proc. Natl. Acad. Sci. U.S.A. 95 (4), 1663–1668. doi: 10.1073/pnas.95.4.1663 9465073 PMC19140

[B12] Zhang F. Hu Z. Wu Z. Lu J. Shi Y. Xu J.. (2021). Reciprocal adaptation of rice and *Xanthomonas oryzae* pv. *oryzae*: cross-species 2D GWAS reveals the underlying genetics. Plant Cell. 33 (8), 2538–2561. doi: 10.1093/plcell/koab146 34467412 PMC8408478

